# Hypertonic Saline Administration via Intraosseous Access During Symptomatic Hyponatremia

**DOI:** 10.7759/cureus.41731

**Published:** 2023-07-11

**Authors:** Angel Juarez, Mitsy Barr, Thaddeus Golden

**Affiliations:** 1 Internal Medicine, Grand Strand Medical Center, Myrtle Beach, USA; 2 Critical Care Medicine, Grand Strand Medical Center, Myrtle Beach, USA

**Keywords:** symptomatic hyponatremia, 3% saline, intravenous vs intraosseous drug administration, syndrome of inappropriate antidiuretic hormone secretion, treatment of hyponatremia

## Abstract

Hyponatremia is a common lab finding. Symptomatology varies greatly and can depend on the degree of hyponatremia and its chronicity. Causes of hyponatremia are also vast and include heart failure, renal injury, liver disease, and gastrointestinal losses, or it can be induced by medication. Treatment depends on the suspected etiology. However, in life-threatening conditions such as seizures or coma, urgent 3% saline is required. Administration of 3% saline is usually through peripheral and central IV access. This case report highlights an alternative route in administering 3% saline, intraosseous vascular access, when other options have been exhausted.

## Introduction

By definition, hyponatremia is defined as serum sodium concentrations of less than 135 mEq/L, which varies depending on the normal values determined at a specific lab. Although the occurrence of hyponatremia is a common electrolyte abnormality, the clinical manifestation varies depending on severity and acuity. At times, it may lead to a life-threatening manifestation. Acute hyponatremia is developed over a period of less than 48 hours, whereas chronic hyponatremia is low serum sodium for 48 hours or more. The degree of hyponatremia can further be classified by serum concentrations. Severe hyponatremia is serum sodium concentrations of <120 mEq/L. Moderate hyponatremia is a serum sodium concentration between 120 to 129 mEq/L, and mild hyponatremia is serum sodium concentrations of 130-134 mEq/L [1-4]. Treatment is dependent on the clinical manifestation. In mild-to-moderate hyponatremia, treatment is generally based on fluid restrictions. Fluid is usually restricted to 1 liter per day or less. In moderate-to-severe cases, pharmacologic therapy is often required. Some of these agents are oral salt tablets, loop diuretics, urea, vasopressin receptor antagonists, or demeclocycline. When neurologic changes are present, such as seizures, obtundation, coma, or respiratory arrest, hypertonic (3%) saline should be used started [1,4,5]. Current recommendations require continuous 3% sodium chloride to be administered through a central catheter, and studies have suggested the possibility of using peripheral lines with a limited infusion-related reaction [6]. In this case report, we present a patient with severe hyponatremia requiring immediate treatment with the inability of obtaining conventional vascular access for 3% saline infusion, highlighting the possibility of intraosseous (IO) access as an equivalent option for central access, in life-threatening conditions.

## Case presentation

**Figure 1:**
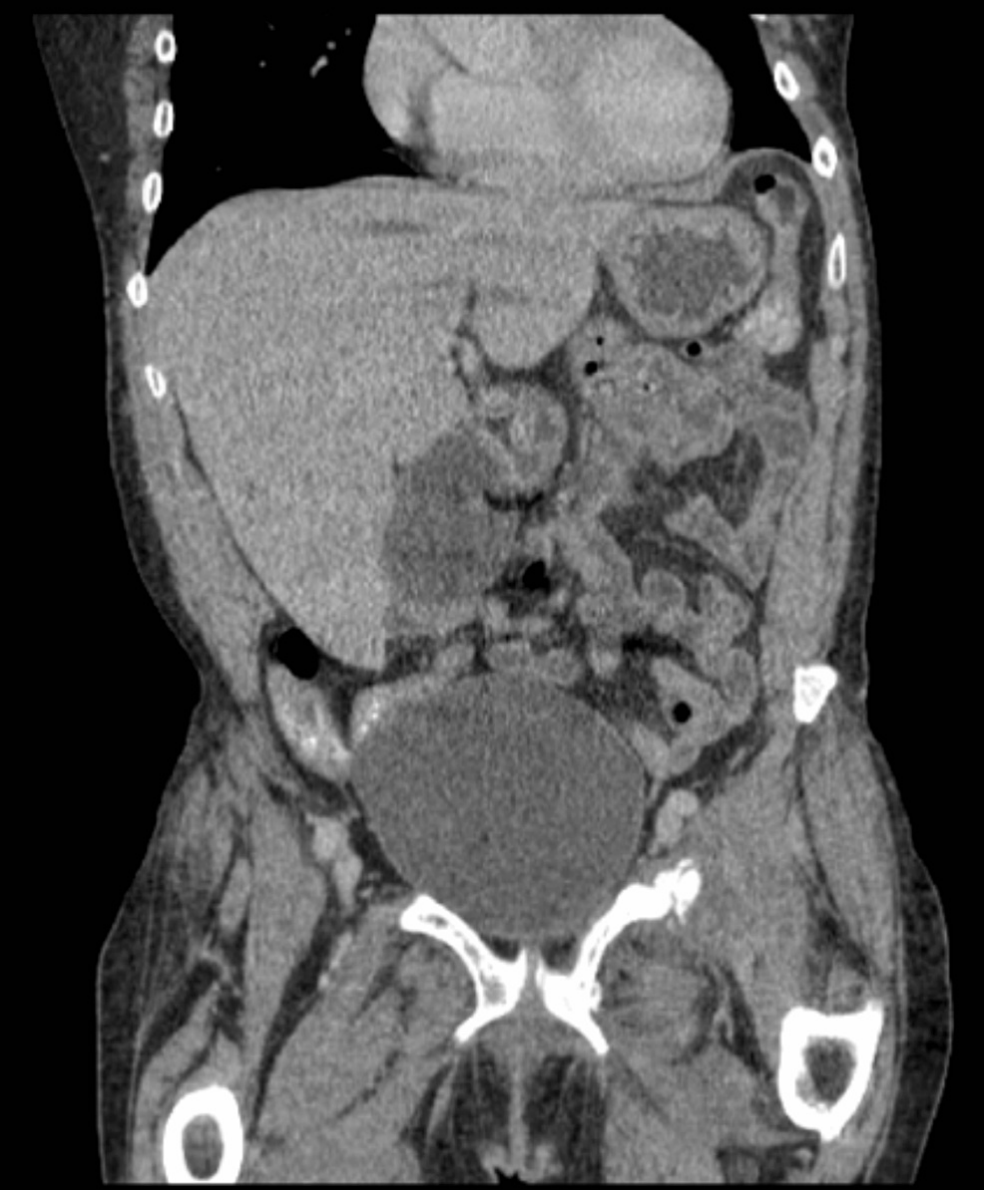
CT of the abdomen and pelvis This CT of the abdomen and pelvis with IV contrast obtained due to the patient's history of loose stool shows a normal liver. Gallbladder mildly distended but no wall thickening. Pancreas is atrophic, and the spleen is normal. Kidneys (not seen in the image) are normal.

A 64-year-old female with a medical history of known chronic hyponatremia secondary to syndrome of inappropriate antidiuretic hormone (SIADH) presented to the emergency department with the chief complaint of loose stool, nausea, and vomiting. The patient denied abdominal pain, chest pain, shortness of breath, weight loss, or changes in urinary habits. On examination, she appeared nauseated, was alert, and was oriented to time, place, self, and situation. Heart and lung sounds were normal. At the time of admission, a significant critical lab of serum sodium of <112 mEq/L was noted. She was given 1 L of normal saline, famotidine 20 mg, and loperamide 2 mg. Two hours after admission, the patient became increasingly agitated. Abdominal CT was normal, as shown in Figure 1. Other imaging studies were unable to be obtained due to the patient’s behavior.

During hospitalization, she continued to endorse nausea and vomiting. Once nausea was controlled with IV antiemetics, she was restarted on her home dose of salt tablets three times a day and continued peripheral normal saline infusion at a rate of 125 cc/hr. Sodium levels improved. Once serum sodium levels reached 121 mEq/L, fluids were stopped, and she continued with salt tablets and remained on a fluid restriction of 1.5 L daily. Unfortunately, the patient, knowingly, began refusing medications and began finding other means of obtaining free water, such as the faucet in her room. Multiple attempts were attempted to inhibit recurrence of symptomatic hyponatremia with signs and patient education. Sodium levels began to decrease once again, causing the patient to become relapse in symptomatology. An attempt was made to start tolvaptan orally. The patient received a dose; however, due to her nausea and vomiting, she was unable to tolerate oral intake, and IV formulation was unavailable at our facility.

On reevaluation, the patient’s mentation continued to decline, with increased confusion, tardive dyskinesia, and nystagmus. Her behavior became disruptive, and peripheral lines were continuously removed by the patient. Because of the patient’s low serum sodium levels of <112 mEq/L, and neurologic findings, the decision was made to infuse hypertonic saline (HTS). Multiple attempts we made by multiple staff members to obtain peripheral access but were unsuccessful. It was determined that the risks outweighed the benefits of placing a central line. As the patient’s mentation continued to worsen, urgent IO vascular access was obtained. An IO needle was inserted into the left proximal tibia and 2% lidocaine was administered prior to infusion. Then, HTS was infused at a rate of 15-20 mL/Hr. After 24 hours, serum sodium was noted to be 121 mEq/L, and mentation improved. Infusion was held at that time, and she was started on tolvaptan 30 mg daily. Two days after discontinuing HTS, and starting tolvaptan, her sodium was noted to be 132 mEq/L, and her mentation returned to her baseline, and she was subsequently discharged.

## Discussion

Treatment and management of hyponatremia is modified based on the underlying pathology. In general, hypovolemic hypo-osmolar hyponatremia can be corrected with isotonic saline, as in the initial presentation of our case. Hypervolemic hypo-osmolar hyponatremia is treated by fluid restriction, diuresis, and, when necessary, dialysis. The mainstay of treatment for euvolemic hyponatremia, as in SIADH, is treated with fluid restriction. However, the use of V2 receptor antagonist or vaptans may be considered as they block the V2 ADH receptor in the collecting duct [7-9]. These medications are used for SIADH.

In severe hyponatremia, as in the presented case, patients may present with various symptoms. In patients with chronic hyponatremia, signs and symptoms may not appear in the acute setting but may appear subtly and progress with time. The recommendations of the U.S. and European guidelines are to treat severe symptomatic hyponatremia with bolus HTS to reverse hyponatremic encephalopathy and increase the serum sodium level by 4 mEq/L to 6 mEq/L within 1 to 2 hours but by no more than 10 mEq/L (correction limit) within the first 24 hours [10]. However, some investigators have a more conservative approach with a correction rate of 0.5 to 1 mEq/L/hr and no more than 4-6 mEq/L in 24 hours. Other authorities recommend up to 8 mEq/L per day but less than 18 mEq/L in 48 hours. [7,8]. In addition to HTS, the use of desmopressin, a synthetic vasopressin receptor agonist, has been controversially used. The literature suggests that it may prevent rapid correction of sodium, whereas others report prolonging recovery times [11]. Desmopressin was not used in our case.

Hypertonic 3% sodium chloride is a hyperosmolar agent used in patients with severe hyponatremic encephalopathy. Many institutions restrict the infusion of 3% sodium chloride to central venous sites due to possible infusion-related adverse effects that may occur with peripheral vascular access. Until recently, studies have reported relatively safe administration through peripheral vein access [6].

As in this case, obtaining vascular access may be difficult and delays patient care in the pre-hospital and hospital setting. IO access is an effective route to give medications and infusions of any type. IO vascular access has been shown by many studies to have a high success rate and is equivalent to central access. Although IO access is superior in many clinical situations, it is underused. Despite the proven value of IO access in critical patients, barriers exist to its use. These barriers include a lack of confidence in the indications for using IO access and the unfamiliarity of staff with IO access. Complications are uncommon and are seen with prolonged use, usually greater than 24 hours. IO infusion allows for blood sampling and administration of virtually all types of fluids and a bioavailability close to the intravenous route [12-17]. In a prospective study of five patients, IO administration of HTS was used in acute brain-injured patients for cerebral edema. In the study, there were no cases of device dislodgement, extravasation, infection, soft tissue injury, or other local complications, suggesting that IO administration of 3% HTS was feasible and well-tolerated [18]. Contraindications of IO access are mainly due to the anatomy of the patient, such as skin infections, fractured bone, severe bone disease, osteogenesis imperfecta, osteoporosis, osteomyelitis, compartment syndrome, prior surgery, and burns [15-17].

In theory, any medication that is administered intravenously may be introduced through IO access. Some key drugs that have been used are amiodarone, atropine, vasopressors (such as dobutamine, dopamine, epinephrine, norepinephrine), propofol, blood products, contrast products, and resuscitative fluids [15]. Due to the safety and effectiveness in the administration of these medications, when vascular access cannot be obtained, as in the presented case, IO access may be an effective alternative.

## Conclusions

We presented a case of hyponatremia, a common condition with various etiologies. In extreme cases, low serum sodium is life-threatening and requires immediate correction. Although current recommendations require continuous 3% sodium chloride to be administered through a central catheter, studies have suggested the possibility of using peripheral lines with a limited infusion-related reaction. In addition to conventional vascular access, the indications of IO vascular infusion in adults are increasing. Studies have initially shown that IO access is not only safe but an effective route of administration of blood products, fluids, and medications of any type. We conclude that IO access may be an effective alternative when conventional vascular access cannot be obtained in urgent cases.
